# Long-term efficacy of drug-coated balloon only angioplasty for IgG4-related coronary artery disease: a case report

**DOI:** 10.1093/ehjcr/ytae492

**Published:** 2024-09-10

**Authors:** Mayu Yamada, Katsumi Ueno, Yoshinobu Kojima, Mitsuru Watanabe, Norihiko Morita

**Affiliations:** Department of Cardiology, Matsunami General Hospital, Kasamatsu, Gifu 501-6062, Japan; Department of Cardiology, Matsunami General Hospital, Kasamatsu, Gifu 501-6062, Japan; Department of Cardiology, Matsunami General Hospital, Kasamatsu, Gifu 501-6062, Japan; Department of Rheumatology, Daido Hospital, 9 Hakusui, Minami, Nagoya City, Aichi 457-8511, Japan; Department of Cardiology, Matsunami General Hospital, Kasamatsu, Gifu 501-6062, Japan

**Keywords:** IgG4-related disease, IgG4-related coronary stenosis, Percutaneous coronary intervention, Drug-coated balloon, Rotational atherectomy, Case report

## Abstract

**Background:**

Although coronary artery involvement in patients with IgG4-related disease (IgG4-RD) is rare, emergency revascularization is recommended for managing acute coronary syndrome. However, coronary aneurysm formation and stent migration after sirolimus-eluting stent implantation have been reported for this disease. Thus, new treatment modalities are warranted for the management of coronary artery disease in this vasculitis.

**Case summary:**

A 70-year-old male who experienced progressive chest discomfort for 1 month underwent cardiac examination. Coronary computed tomography angiography (CCTA) revealed right coronary artery hypoplasia, coronary artery aneurysm with severely calcified stenosis in the proximal left anterior descending artery (LAD), and subtotal obstruction in the mid-LAD. The left circumflex artery (LCX) also had stenosis and dilated lesions. Additionally, diffuse perivascular soft tissue thickening was observed in the mid-LCX. The percutaneous coronary intervention was performed for the proximal- and mid-LAD lesions. Rotational atherectomy with low-pressure drug-coated balloon (DCB) dilation was considered for these lesions owing to suspicion of vasculitis. The patient was later diagnosed with Sjögren’s syndrome and IgG4-RD overlap syndrome. Oral steroids and immunosuppressive drugs were initiated. In a follow-up at 7 and 26 months, late lumen enlargement was observed in the treated area of the LAD, without enlargement of the adjacent aneurysm. CCTA performed after 26 months revealed resolution of the diffuse perivascular soft tissue thickening in the mid-LCX.

**Discussion:**

To our knowledge, this is the first case report demonstrating favourable outcomes for treatment of a coronary artery lesion attributed to IgG4-RD with DCB, leaving nothing implanted in the coronary artery tree.

**Learning points**

- Coronary artery involvement in IgG4-related disease (IgG4-RD) is rare, and no appropriate treatment has been established.
- Coronary artery lesions attributed to IgG4-RD were treated with a drug-coated balloon (DCB), resulting in good long-term outcomes.
- Systemic therapy may be considered effective for managing coronary artery lesions; however, DCB should be considered in cases requiring urgent percutaneous coronary intervention.

## Introduction

IgG4-related disease (IgG4-RD) is a systemic chronic lymphoproliferative disease characterized by IgG4-positive plasma cell infiltration and fibrosis in various organs. Although periaortitis, coronary arteritis, and pericarditis are typical cardiovascular manifestations of IgG4-RD, coronary artery disease (CAD) is rare,^1,2^ and specific characteristics and effective treatment are yet to be clearly elucidated.

Few reports on revascularization therapy for this disease are available. Moreover, stent migration and occlusion caused by progression of coronary aneurysm have been reported 9 months after sirolimus-eluting stent implantation for a patient with CAD in IgG4-RD.^3^ Therefore, new treatment modalities for IgG4-RD-CAD are needed. Drug-coated balloon (DCB) is one of the leading-edge devices that transfer antiproliferative drugs into the lesions via single balloon inflation to prevent restenosis, leaving nothing implanted in the coronary artery tree. In this report, we describe a case with CAD in IgG4-RD that responded favourably to local treatment with DCB and immunosuppressive agents.

## Summary figure

**Table:** 

Time	Event
**June 2021**	The patient became aware of chest tightness on exertion.
**July 2021**	The patient was admitted for a transient cerebral ischemic attack and had bilateral internal carotid artery stents implanted. At that time, an abnormal electrocardiogram was observed.
**October 2021**	Coronary computed tomography angiography (CCTA) and coronary angiography (CAG) revealed severe three-vessel disease with coronary artery aneurysm and stenosis. Based on the findings, we suspected vasculitis and treated the left anterior descending artery (LAD) with drug-coated balloon (DCB)-only angioplasty after debulking with a rotablator.
**November 2021**	Based on the blood tests and biopsy results, the patient was determined to have Sjögren's syndrome and IgG4-related disease overlap syndrome by a rheumatologist.
**January 2022**	The patient was hospitalised for interstitial pneumonia. He underwent steroid pulse therapy and was then started on oral immunosuppressive drugs and steroids.
**May 2023**	In the CAG at 7 months, late lumen enlargement was observed in the treated lesions of the LAD; however, no enlargement of the aneurysm was observed.
**February 2023**	CAG after 26 months showed good patency of the LAD and spontaneous regression of the stenosis in the left circumflex artery (LCX). The CCTA revealed loss of soft tissue thickening around the coronary artery lesion in the central part of the LCX.

## Case presentation

A 70-year-old man, experiencing chest tightness during exertion for 1 month, was admitted to the hospital with a transient cerebral ischaemic attack. Following urgent bilateral carotid artery stenting, his chest pain progressively occurred even at rest.

**Figure 1:**
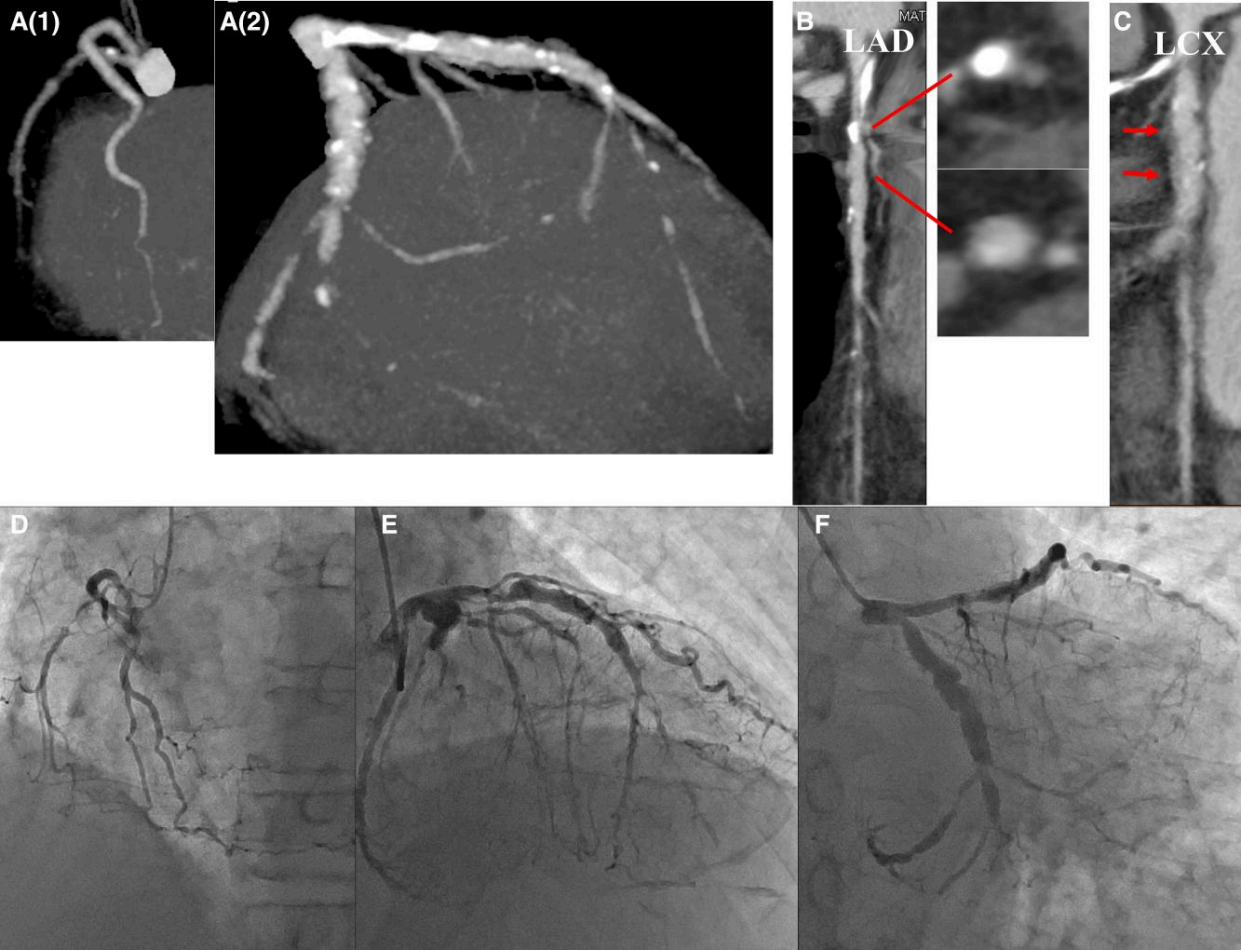
Coronary artery imaging. Coronary computed tomography angiography shows hypoplasia in the right coronary artery (*A*1) and a mixture of dilated lesions and stenosis in the left coronary artery (*A*2). (*B*) The short axes of the calcified lesion and coronary aneurysm in the left anterior descending artery are shown (arrows). (*C*) In the left circumflex artery, diffuse perivascular soft tissue thickening is observed (arrows). Coronary angiography shows a severe three-vessel disease with aneurysmally dilated lesions and stenosis (*D*, *E*, *F*).

The patient was a current smoker (20 cigarettes/day for 50 years) with a history of hypertension and dyslipidaemia. Low-density lipoprotein-cholesterol was well controlled at 64 mg/dL (normal: 65–163) with atorvastatin 10 mg/day. No remarkable findings were noted upon physical examination, and transthoracic echocardiography revealed normal left ventricle function in spite of abnormal Q waves in the II, III, and aVF leads of the electrocardiogram. Coronary computed tomography angiography (CCTA) revealed right coronary artery hypoplasia, coronary artery aneurysm with severely calcified stenosis in the proximal left anterior descending artery (LAD) and subtotal obstruction in the mid-LAD. The left circumflex artery (LCX) showed significant stenosis in the ostium, aneurysmally dilated lesions in the mid and total occlusion in the distal segment. Moreover, diffuse perivascular soft tissue thickening was detected in the mid-LCX. Additional coronary angiography (CAG) also revealed a severe three-vessel disease with aneurysmally dilated lesions both in the LAD and LCX (*Figure 1*, Video 1).

**Figure 2:**
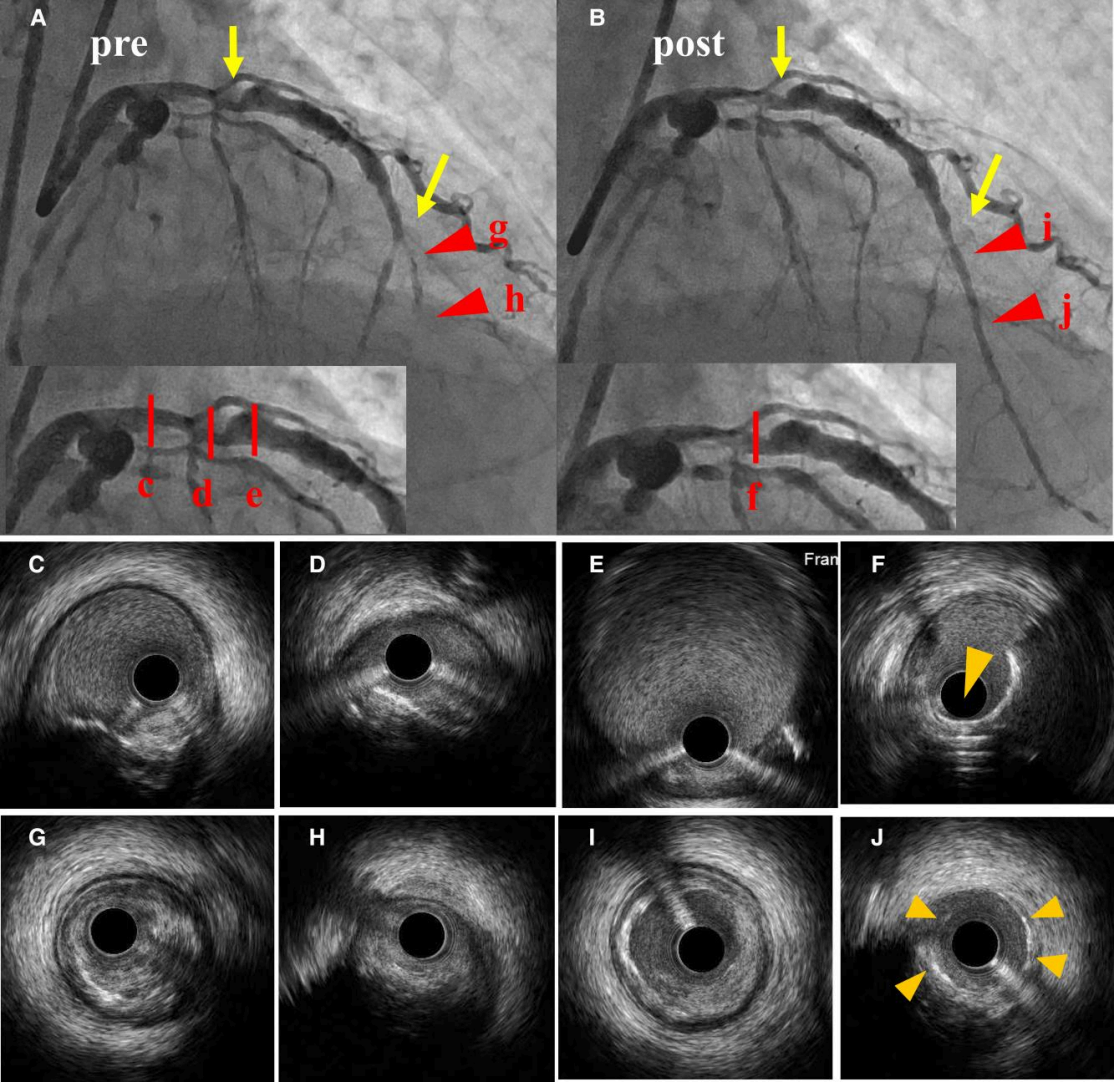
Percutaneous coronary intervention in the left anterior descending artery. Percutaneous coronary intervention (PCI). Initial (*A*) and final angiography after PCI (*B*) are shown. The target lesions are indicated by arrows. Intravascular ultrasound (IVUS) images reveal the proximal portion of the stenosis (*C*), proximal target stenosis with calcification (*D*), and the distal coronary aneurysm (*E*). (*F*) The final IVUS image after rotablation with a 2.0 mm burr (175 000 rpm) followed by drug-coated balloon dilatation, shows that the calcification has been effectively removed (arrowheads). (*G*), (*H*) The distal lesion before PCI. (*I*), (*J*) The final IVUS image after rotablation with a 1.5 mm burr (175 000 rpm) followed by drug-coated balloon dilatation, shows that the calcification and the hard fibrous plaque have been ablated. Image of a vessel wall with drug application (arrowheads).

As both the distal runoff of the LAD and LCX were too poor for grafting under bypass surgery, percutaneous coronary intervention (PCI) was performed for the culprit proximal- and mid-LAD lesions with aspirin 100 mg/day and clopidogrel 75 mg/day. DCB-only angioplasty was considered for these lesions as the patient was suspected of having vasculitis owing to the specific aneurysmal appearance of the coronary artery and presence of diffuse perivascular soft tissue thickening in the LCX. PCI was performed via the right femoral artery. The mid-subtotal lesion in the LAD could not be fully dilated with a 1.5 mm balloon and an intravascular ultrasound (IVUS) imaging demonstrated diffuse fibrosis and fibro-calcified lesions in the distal LAD. Thus, rotational atherectomy for both the proximal and mid-lesion was performed with a 1.5 mm burr (175 000 rpm) (Boston Scientific, USA), followed by debulking of the proximal lesion with a 2.0 mm burr (175 000 rpm). Additional scoring balloon dilatation [Lacrosse NSE® 2.5 mm at 4atm (Nipro, Japan)] for the mid-LAD and DCB angioplasty [SeQuent Please® 2.5 mm at 2atm for the mid-lesion and SeQuent Please® 3.5 mm at 2atm for the proximal lesion (B. Braun, Germany)] was performed, and a smooth lumen without coronary dissections was obtained (*Figure 2*) (see Supplementary material online, *Videos S1* and *S2*). The patient’s symptoms resolved, and he was discharged.

Blood test results showed positive antinuclear antibodies 320× (normal < 40×), anti-ds-DNA antibodies 196 IU/mL (normal < 12), and anti-SS-A/anti-SS-B antibodies, with positive lymphocytic infiltration in the labial salivary gland histopathology leading to the diagnosis of Sjögren’s syndrome (ACR/EULAR classification criteria).^4^ The patient also exhibited elevated IgG (1792 mg/dL; normal: 861–1747) and IgG4 (230 mg/dL; normal: 11–121) levels. Because Sjögren’s syndrome is not presumed to be complicated by vasculitis, the rheumatologist diagnosed this case as an overlap between Sjögren’s syndrome and IgG4-RD. Three months after revascularization, the patient developed interstitial pneumonia and was started on oral steroids and immunosuppressive drugs. He is currently undergoing treatment with prednisolone 3 mg/day, tacrolimus 3 mg/day, nintedanib 150 mg/day, abatacept 125 mg/day, and clopidogrel 75 mg/day; his IgG4 levels are normal.

**Figure 3:**
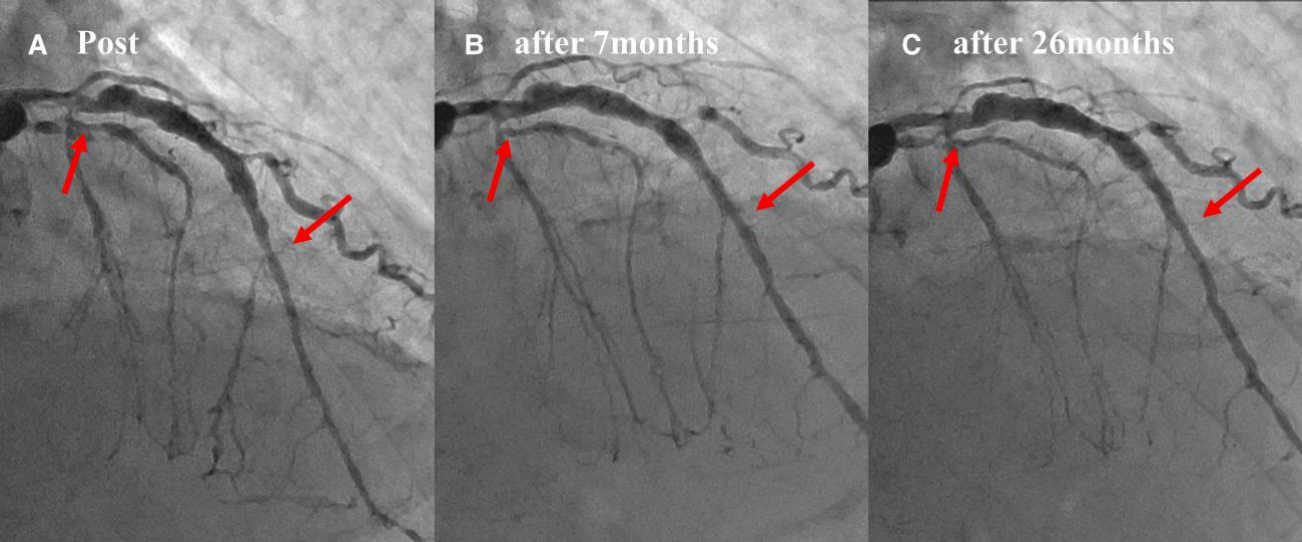
Serial angiographical changes of the treated lesions. Serial angiographical changes of the treated lesions (arrows) are shown. (*A*) Post-intervention. (*B*) Seven months after intervention. The diameters of both the proximal and distal treated lesion are enlarged compared with those of the post-intervention (late lumen enlargement). (*C*) Twenty-six months after intervention. The lumen of the treated lesions is maintained without any changes in the size of the aneurysm.

**Figure 4:**
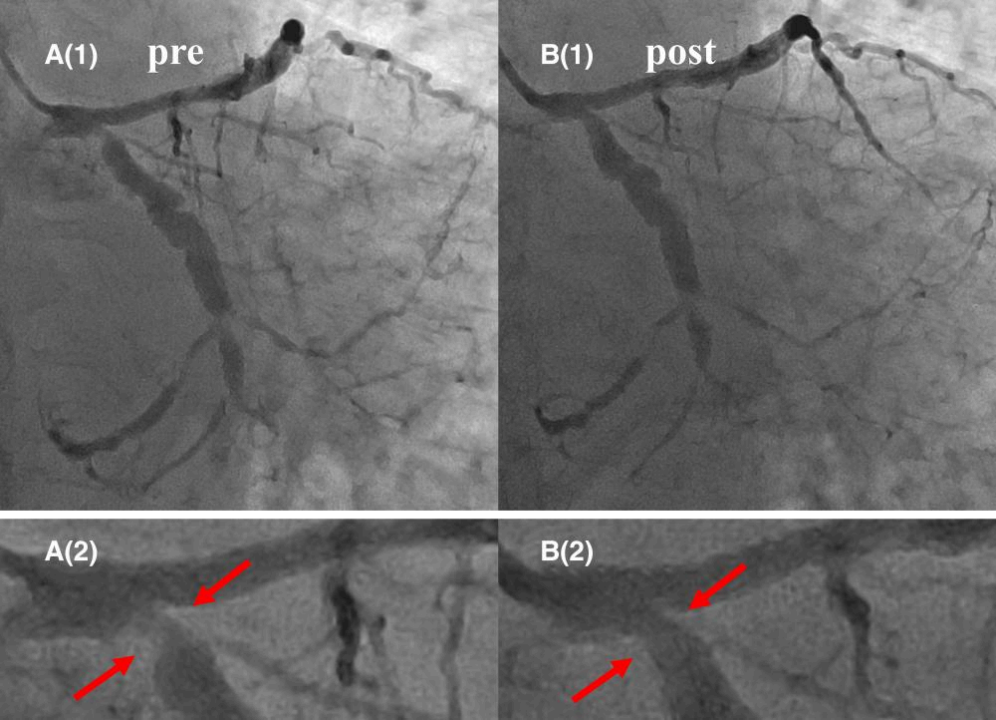
Natural regression of the ostial lesions in the left circumflex artery. Natural regression of the ostial lesions in the left circumflex artery is shown (arrows). (*A*1) Initial coronary angiography. (*B*1) Twenty-six months later. Magnified images are shown in *A*2 and *B*2.

**Figure 5:**
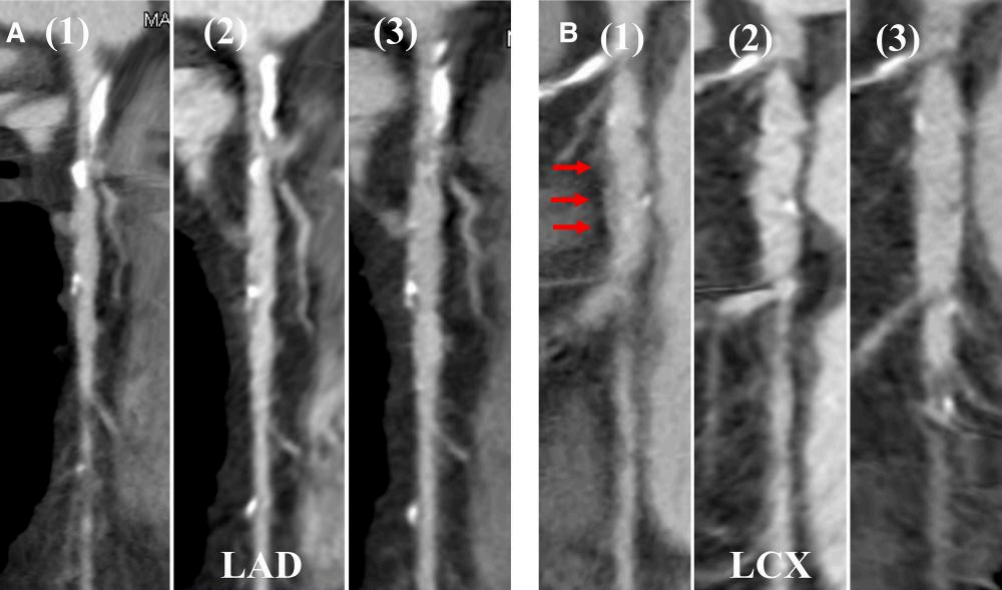
Serial changes in coronary computed tomography angiography of the left coronary artery. Serial changes of coronary computed tomography angiography findings of the left coronary artery are shown. (*A*) The left anterior descending artery. (*A*1) Pre-intervention. (*A*2) Fourteen months later. (*A*3) Twenty-six months later. Note no change in the size of the aneurysm. (*B*) Left circumflex artery. The arrows in (*B*1) show diffuse perivascular soft tissue thickening, which gradually disappeared after 14 (*B*2) and 26 (*B*3) months.

CAG performed at 7 months showed late lumen enlargement (LLE) in both the treated areas of the LAD without restenosis, and no enlargement of the aneurysm was observed (*Figure 3*, Video 2). After 26 months, a repeat CAG showed that the treated lesions in the LAD remained open, and natural regression of the ostial lesions in the LCX was observed (*Figure 4*, Video 3). Furthermore, CCTA performed after 26 months revealed a lack of diffuse perivascular soft tissue thickening in the mid-LCX (*Figure 5*).

## Discussion

A total of 23.9% of patients with IgG4-associated pericoronary arteritis demonstrated isolated pericoronary arteritis without the involvement of other organs, with significantly lower IgG4 antibody titre. Mass-like or diffuse wall thickening, which was the most frequently observed type (78.6%), can be considered early-stage lesions that progress to aneurysms or stenotic lesions.^5^ In this case report, the IgG4 antibody titre was relatively low at 230 mg/dL (normal: 11–121), and diffuse perivascular soft tissue thickening, aneurysm, and stenosis were observed (*Figure 1*).

CAD in IgG4-RD can cause serious cardiovascular events, such as ruptured aneurysms and acute coronary syndrome.^6–8^ Thus, the possibility of CAD in IgG4-RD should be considered based on the clinical symptoms and imaging findings. However, no solid evidence exists for its treatment. The use of stents is not always possible or desirable due to chronic inflammation problems attributed to foreign-body placement and anatomical challenges in implantation.^9^ Moreover, for severely calcified lesions of CAD in IgG4-RD, high-pressure balloon inflation carries a risk of stent overexpansion, neointimal overgrowth, and aneurysm formation. Therefore, a less traumatic treatment was performed.^10^ Although improvement in coronary artery stenosis and resolution of pericoronary soft tissue lesions with immunosuppressive agents alone have been reported,^5,11,12^ one cannot await the effects of drug therapy in emergency cases, such as acute coronary syndrome.

To our knowledge, this is the first case report of CAD in IgG4-RD treated with a combination of rotational atherectomy and low-pressure DCB dilation with favourable long-term outcomes. The lesion exhibited good patency after 26 months, and the patient also demonstrated LLE, which is often observed in the late follow-up of DCB angioplasty without aneurysmal enlargement.^13,14^ Recent studies demonstrated the safety and long-term efficacy of DCB-only angioplasty for large vessel or calcified lesions as well as small vessel CAD.^10^ Paclitaxel applied to DCB is known to inhibit proliferation of the neointima and smooth muscle cells, which inhibits cell division *in vitro* by blocking the microtubules.^9^ This reaction persists only for approximately 4 weeks in the tissue. We hypothesized that paclitaxel may also inhibit the proliferation of mitotic IgG4-positive plasma cells and that it may act locally on the IgG4-positive plasma cells clustered in the coronary arteries.

The long-term success of DCB angioplasty in this case may encourage the use of DCB for the treatment of CAD in IgG4-RD. Furthermore, the ostial lesion in the LCX regressed naturally (*Figures 4* and *5*), indicating the need for systemic drug therapy. In conclusion, patients with a characteristic coronary artery picture, such as coronary aneurysm or stenosis, should be suspected of having vasculitis, and DCB treatment can be considered a viable treatment option.

## Lead author biography


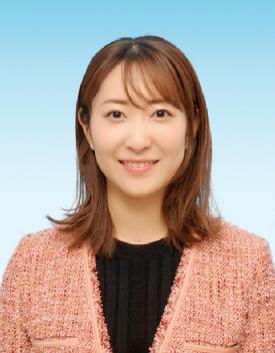
I was born in Yokohama, Japan, in 1993. I received my MD degree from Saitama Medical University, Japan, in 2018. I completed 2 years of the Japanese residential programme at Saitama Medical University International Medical Center (2018=E2=80=932020). I worked in the Department of Cardiology at the Gifu Heart Center and am now working in the Department of Cardiology at Matsunami General Hospital.

## Supplementary Material

ytae492_Supplementary_Data

## Data Availability

All data regarding this case are presented within the manuscript and supplementary material.
